# Supplementary material for: A Rice R2R3-Type MYB Transcription Factor OsFLP Positively Regulates Drought Stress Response via OsNAC
Source: Int J Mol Sci. 2022 May 24;23(11):5873. doi: 10.3390/ijms23115873 (PMC9180394; doi:10.3390/ijms23115873)
Supplement: Supplementary file 1 [file ijms-23-05873-s001.zip › ijms-1711841-supplementary.pdf]

Table S1. Cis-acting elements in the promoter region of *OsNAC1* and *OsNAC6* binding by MYB transcription factor.

Matrix ID	Score	Position	Strand	Sequence (CAPITALS: core sequences)
OsNAC1 NC_029258.1	8.491339	-551 to -542	+	CAACCTAAGA
	6.766874	-699 to -690	+	AAACCTTAAA
	6.0062394	-1208 to 1219	-	AAACCTCAAG
	5.913508	-623 to -614	+	TGTCCTAATT
	5.6907196	-1361 to -1352	+	AAAACTAAAA
	5.5058193	-307 to -298	+	TGACCTCGGC
	4.659336	-698 to -689	+	AACCTTAAAA
	4.070128	-782 to -773	-	TAAACTATTT
	3.9510279	-1052 to -1043	-	TACTCTACTT
	3.7640855	-1779 to -1770	+	AAACCCAACC
	3.6692517	-1778 to -1769	+	AACCCAACCA
OsNAC6 NC_029256.1	8.695591	-1332 to -1323	+	AGACCTACTT
	6.7416577	-1200 to -1191	+	GACCCTACAG
	6.2968206	-1054 to 1495	-	ACACCTAAGC
	6.090226	-86 to -77	+	AACACTAGTA
	5.926167	-872 to -863	-	AGCCGTAGCT
	5.786418	-797 to -788	-	TGACATAGAA
	5.460014	-1021 to -1012	-	AAACCTTCTT
	5.359429	-745 to -736	-	AACTCTAAAC
	5.177283	-1515 to -1506	+	TCCCCTACGA
	5.139875	-1361 to -1352	+	TACCCAAAAA
	5.0971913	-300 to -291	+	TGACGTAAGC
	4.9563427	-889 to -880	-	TGCTCTAAAT
	4.770077	-81 to -72	-	TATCCTACTA
	4.5864944	-106 to -97	+	AGCCCTCCTC
	4.4865985	-1999 to -1990	-	TGATCTAACT
	4.3426924	-1764 to -1755	-	ATCCCTTATA
	4.3426924	-1580 to -1571	+	ATCCCTTATA
	4.1384006	-394 to -385	+	CACCCTGCGG
	3.942666	-814 to -805	+	TTACCTTACA

	3.8799586	-473 to -464	+	TTCCCTCGCC
	3.7542815	-1220 to -1211	-	CGACCTCACA
	3.6927981	-1816 to -1807	-	ATTCCTAATT
	3.487473	-1141 to -1132	+	AAATCTACAC

Table S2. Primers were used in this study.

Primer name	Forward sequence (5′-3′)	Reverse sequence (5′-3′)	Purpose
OsACTIN	GGATCCATCTTGGCATCTCTCA	GGGCCAGACTCGTCGTACTC	qRT-PCR
OsFLP	AGTTCTTGGGAACAAATGGAC	TGTACCAATGCCCTCTTTGA	qRT-PCR
OsNAC1	CATGGTCCCGTTCTGAGGTG	CACACGTTGCAGCATCGATC	qRT-PCR
OsNAC4	GGTGAAGGAGGACAACGACT	TCAGAATGGTGGCAGGATTGT	qRT-PCR
OsNAC5	GGTTGGATGATTGGGTGTTGT	CGTAGAACCCATAGTCGGAGA	qRT-PCR
OsNAC6	CTGTACGGAGAGAAGGAGTGG	GTGCATGATCCAGTTGGTCT	qRT-PCR
OsDST	ATCCAAGAAGGCAAGGTCAATC	CACACGAGGAGGAATTGGAAG	qRT-PCR
Osperoxidase 24 precursor	GTCTCCAGGACCTCGTCGTC	AAAAGGTTGCAGTGCCCG	qRT-PCR
OsLEA3	CGGCAGCGTCCTCCAAC	CGGTCATCCCCAGCGTG	qRT-PCR
OsDREB2A	GCTGCACATCAGCACCTTCA	TCCTGCACCTCAGGGACTAC	qRT-PCR
proOsNAC1	GGAATTCCACACATCTCGCATGG-TAAC	GCGTCGACCGCTTCTTGCTT-GCTTCTC	Y1H
proOsNAC4	GGGTACCGGTGTCACATCGGA-TATTTG	CAGCCTCTGACAAAGCAGTC	Y1H
proOsNAC5	GGAATTCCTATTGTCAC-GAGCCAAGG	GGGTAC-CTCAATCTCCGATCTGAGGCC	Y1H
proOsNAC6	GGAATTCCAATGTTGAC-GGGTCAATGAG	CCTCGAGCTCTCTCCCCCTTCTCCGGT	Y1H
OsFLP-RNAi	GCGTCGACCCCGGGTGTGCTTCG-TACTCAAATTGC	GGGGATCCGAGCTCAG-TTCTGCCTGAGACAACCTT	Knockdown line generation
OsFLP-OE	ATGGCGACCGGACCCGATCTG	TCATAGGCTATTAAGGAGAGC	Over-expression line generation

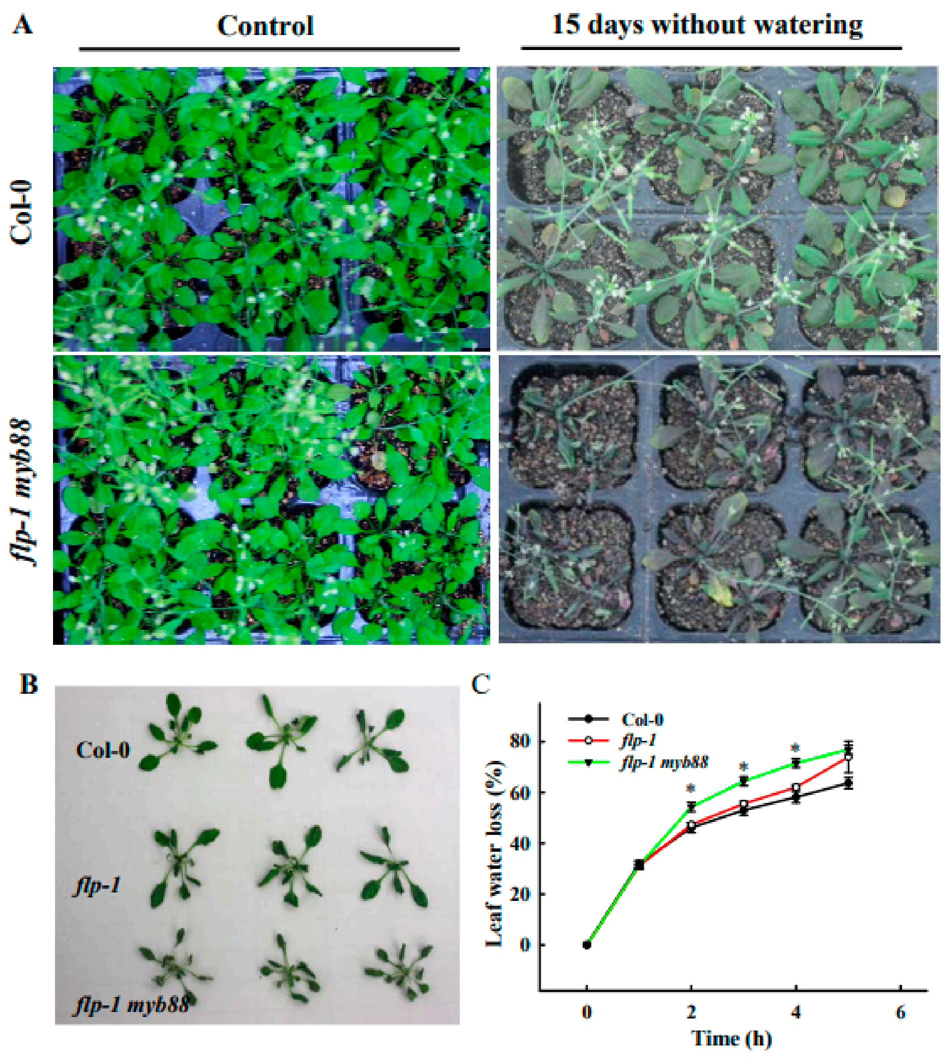

Figure S1. Arabidopsis flp-1 myb88 double mutants were sensitive to drought stress. (A) Drought sensitivity was rescued in flp-1 myb88 mutants compared with Col-0 after 15 days without water. (B,C) Water loss were detected from detached leaves. Asterisks indicate a significant difference flp-1 myb88 mutants to Col-0. (Student's t-test, *$P < 0.05$).

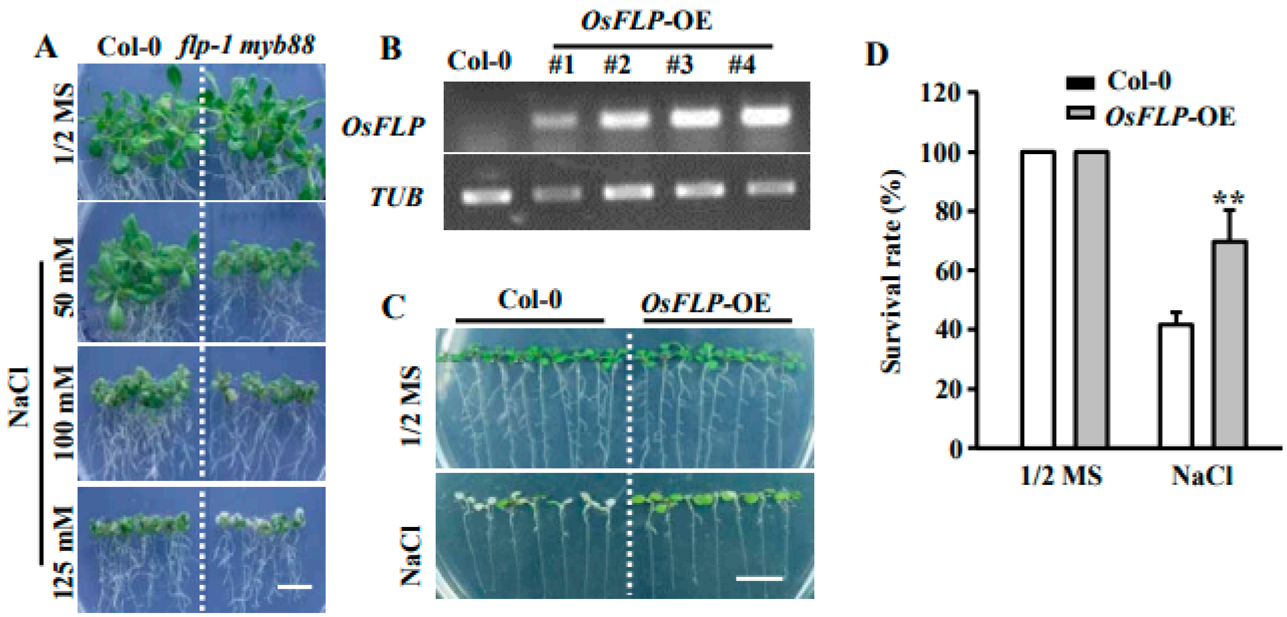

Figure S2. Overexpression of OsFLP enhanced salt tolerance both in rice and Arabodpsis. (A) Five days Col-0 and flp myb88 seedlings were treated by 50 mM 100 mM and 125 mM NaCl for one week. Bars = 1.0 cm. (B) Expression levels of OsFLP overexpression lines were detected by semi-quantitative reverse transcription PCR (SqRT-PCR). (C) Five days Col-0 and OsFLP seedlings were treated by 150 mM NaCl for one week. Bars = 1.0 cm. (D) Analysis of survival rate after salt treatment. Error bar means ± SD. Asterisks indicate a significant difference to wild type (Student's t-test, **P < 0.01, *P < 0.05).

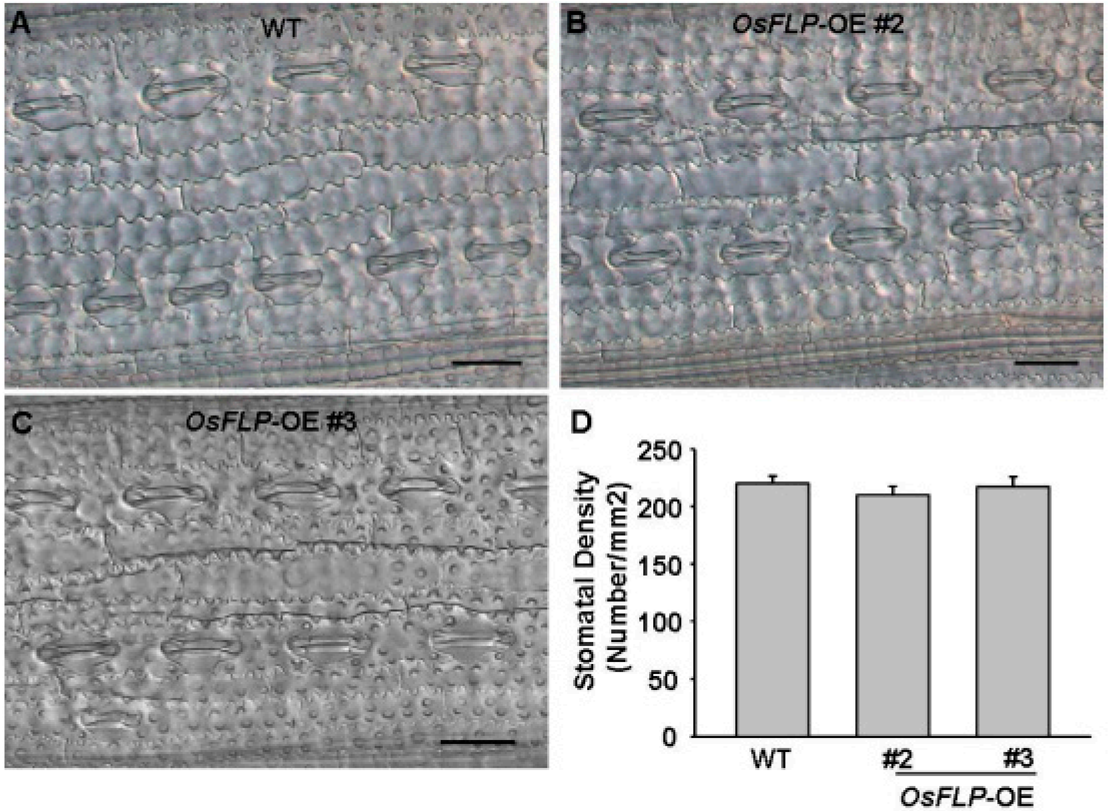

Figure S3. Overexpression of OsFLP gene has no obvious impact on stomatal density. (A-C) DIC images of rice leaf epidermis from 10-day-old WT and OsFLP-OE plants. Bars = 20 µm. (D) No significant difference of stomatal density was found between OsFLP-OE and WT mature leaves (n = 10).

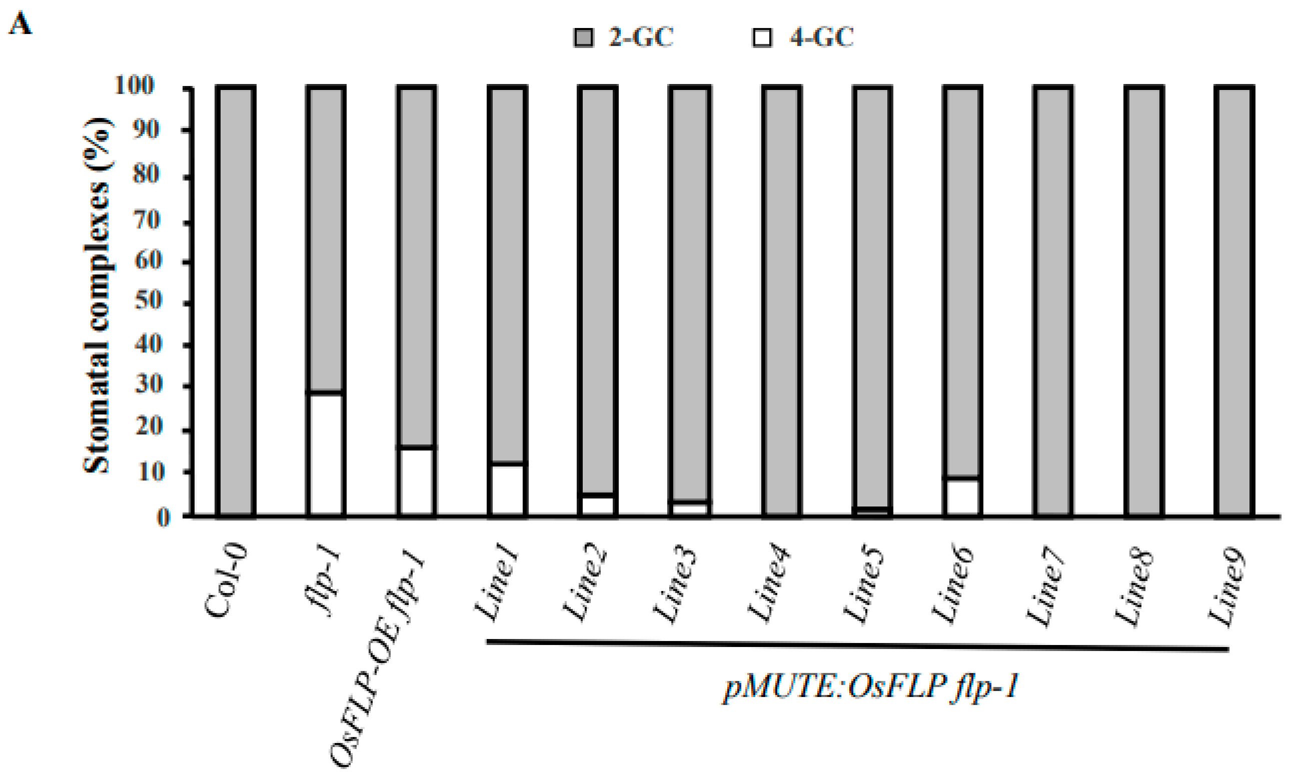

Figure S4. Ectopic expression of OsFLP rescue the stomatal cluster of flp-1 mutant. (A) Quantification of effects of 35S:OsFLP and pMUTE:OsFLP on flp-1 stomatal phenotypes. Stomata from 10-20 cotyledons were scored for normal and cluster stomatal phenotypes. Grey, normal 2-GC; White, cluster stomata with 4 GCs.

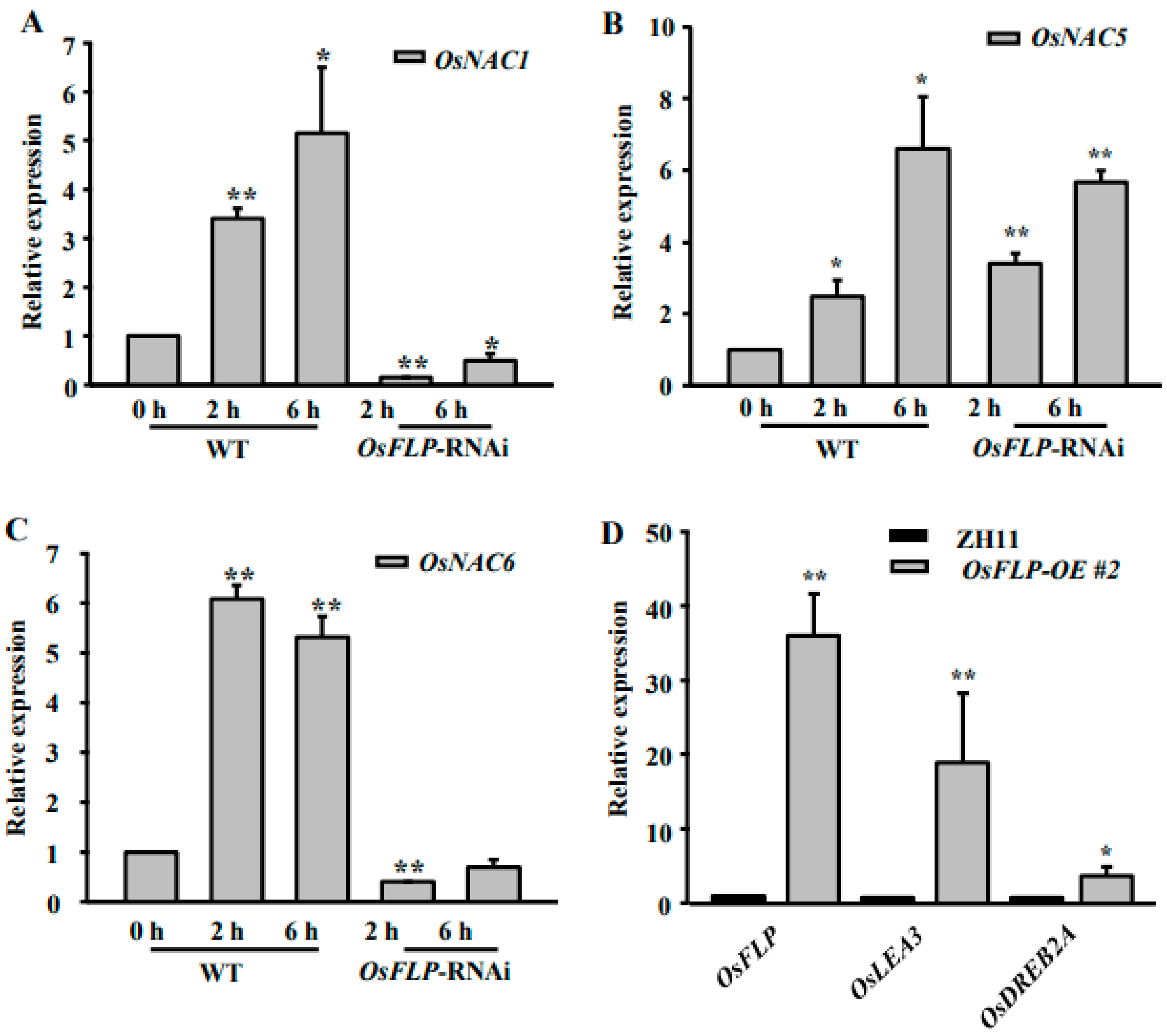

Figure S5. Expression levels of NAC genes were reduced in OsFLP-RNAi transgenic rice plants. The relative expression levels of OsNAC1 (A), OsNAC5 (B), OsNAC6 (C), and stress response genes OsLEA3 and OsDREB2A (D) were assessed using qRT-PCR. Error bars represent SD of three biological replicates (Student's t-test, $^{**}P < 0.01$, $^{*}P < 0.05$).
